# Cost-effectiveness of long term left ventricular assist devices

**DOI:** 10.1007/s12471-024-01909-0

**Published:** 2024-11-06

**Authors:** Gerardus P. J. van Hout, Pieter A. Doevendans, Linda W. van Laake

**Affiliations:** 1 Department of Cardiology, St. Antonius Hospital, Nieuwegein, The Netherlands; 2 Department of Cardiology, University Medical Center Utrecht, Utrecht, The Netherlands; 3 Netherlands Heart Institute, Utrecht, The Netherlands; 4 Central Military Hospital, Utrecht, The Netherlands

Dear Editor,

With great interest we read the systematic review by Wiethoff et al. regarding the cost-effectiveness of therapies for inherited cardiomyopathies [1].

Left ventricular assist devices (LVADs) have demonstrated a significant survival benefit in patients with end stage heart failure (HF). To our surprise, the infographic (Fig. 1) in their review suggests that LVADs are not cost-effective in patients with dilated cardiomyopathy. Although these assertions are nuanced in the accompanying text, we believe the information in this central figure is not correct.

Wiethoff et al. based their conclusions on two papers. These papers either investigate the usage of (Bi)VADs in children or analyse the cost-effectiveness of mechanical support with earlier-generation LVADs in Japan, which have never been available on the European market [2].

In our opinion, these key differences render their analysis non-contributory and non-extrapolatable for assessing the cost-effectiveness of LVAD therapy in adult HF patients in Europe. Thorough research on this subject has been performed in a similar health care system showing cost-effectiveness with the newest generation LVADs for end-stage HF [3].
